# APPLYING THE DIME APPROACH TO A FINANCIAL LITERACY PROGRAM

**DOI:** 10.1093/geroni/igz038.2152

**Published:** 2019-11-08

**Authors:** Lisa M Brown

**Affiliations:** Palo Alto University, Plo Alto, California, United States

## Abstract

Financial literacy is defined as “the ability to use knowledge and skills to manage financial resources effectively for a lifetime of financial well-being.” The Money Smart Peer-to-Peer Program (MSP) was developed by the FDIC for use with diverse populations. MSP was culturally adapted for use in Jamaica using the Johns Hopkins DIME process. Peer counselling programs consistently demonstrate that they are acceptable and effective with adults who have limited education and low-income by overcoming barriers that are typically encountered when people attempt to access unfamiliar services or guidance from a professional. Research conducted with the MSP program reveals that program participants were more likely to open and save using deposit accounts, use and adhere to a budget, and have increased confidence in their financial abilities 6 to 12 months after completing the course. Implementation of MSP increases the likelihood that people can retire with dignity and financial security.

